# The Use of ß-Elemene to Enhance Radio Sensitization of A375 Human Melanoma Cells

**DOI:** 10.22074/cellj.2020.6326

**Published:** 2019-07-31

**Authors:** Zahra Balavandi, Ali Neshasteh-Riz, Fereshteh Koosha, Samira Eynali, Mahmood Hoormand, Minoo Shahidi

**Affiliations:** 1 Radiation Biology Research Center, Iran University of Medical Sciences, Tehran, Iran; 2 Department of Radiation Sciences, School of Allied Medical Sciences, Iran University of Medical Sciences, Tehran, Iran; 3 Department of Medical Physics and Biomedical Engineering, School of Medicine, Tehran University of Medical Sciences, Tehran, Iran; 4 Department of Pharmacology, School of Medicine, Iran University of Medical Sciences, Tehran, Iran; 5 Department of Hematology, School of Allied Medical Sciences, Iran University of Medical Sciences, Tehran, Iran

**Keywords:** Apoptosis, Beta-Elemene, Melanoma, X-ray

## Abstract

**Objective:**

Melanoma is the most malignant and severe type of skin cancer. It is a tumor with a high risk of metastasis and resistant to conventional treatment methods (surgery, radiotherapy, and chemotherapy). β-elemene is the most active constituent of Curcuma wenyujin which is a non-cytotoxic antitumor drug, proved to be effective in different types of cancers. The study aimed to investigate the therapeutic effects of β-elemene in combination with radiotherapy on A375 human melanoma.

**Materials and Methods:**

In this experimental study, human melanoma cells were grown in the monolayer culture model. The procedure of the treatment was performed by the addition of different concentrations of β-elemene to the cells. Then, the cells were exposed to 2 and 4 Gy X-ray in different incubation times (24, 48, and 72 hours). The MTT assay was used for the determination of the cell viability. To study the rate of apoptosis response to treatments, the Annexin V/PI assay was carried out.

**Results:**

The results of the MTT assay showed β-elemene reduced the cell proliferation in dose- and time-dependent manners in cells exposed to radiation. Flow cytometry analysis indicated that β-elemene was effective in the induction of apoptosis. Furthermore, the combination treatment with radiation remarkably decreased the cells proliferation ability and also enhanced apoptosis. For example, cell viability in a group exposed to 40 µg/ml of β-elemene was 80%, but combination treatment with 6 MV X beam at a dose of 2 Gy reduced the viability to 61%.

**Conclusion:**

Our results showed that β-elemene reduced the proliferation of human melanoma cancer cell through apoptosis. Also, the results demonstrated that the radio sensitivity of A375 cell line was significantly enhanced by β-elemene. The findings of this study indicated the efficiency of β-elemene in treating melanoma cells and the necessity for further research in this field.

## Introduction

Melanoma is the most malignant and severe skin cancer type. It is a tumor with a risk of high metastasis and accounts for 75 % of deaths associated with skin cancer (1). This type of skin cancer is rapidly growing in recent years, and this can be due to chronic exposure of skin to sun rays without the use of equipment for the protection against sunlight, which especially can lead to melanoma in Caucasians (2). Patients suffering from melanoma may undergo surgery and/ or receive chemotherapeutic agents and radiotherapy or receive a combination of these treatments (3). In addition, metastasis of the tumor is a significant problem following surgery (4). Chemotherapy drugs are often used for the treatment of melanoma include cisplatin and dacarbazine. Despite the efficacy of these therapies, several adverse effects have been so far reported such as tumor resistance to medications and cytotoxicity such as ototoxicity, nephrotoxicity, and leucopenia (5). Radiotherapy can be applied following surgery and chemotherapy considering the depth of the lesion and the severity of the disease. The irradiation could be performed by photon or electron. Usually, the dose range between 1.8 to 2 (Gy) could be employed per fraction. Melanoma tumors are among the most resistant cells to radiation (6). Radiosensitizer drugs have been developed to reduce the dose of radiation and the side effects of radiotherapy with the same outcomes (7). 

Elemene is a compound extracted from Curcuma wenyujin which, for the first time, was used against cancer in China (8). ß-elemene is the active component of Curcuma wenyujin that is a non-cytotoxic antitumor drug (9). Recent studies have shown that ß-elemene may sensitize tumor cells to chemotherapy drugs such as cisplatin, taxanes, and paclitaxel (10-12). As reported in previous studies, treatment with ß-elemene is useful for the treatment of leukemia, HCC, glioblastoma, breast, bladder, lung, gastric, prostate, ovarian, and liver cancers (13-16). The beneficial effect of ß-elemene such as low toxicity, low side effects, well tolerance by patients, high potency, and high synergistic effects with other anti-tumor drugs have made ß-elemene a bona fide candidate for the treatment of various type of cancers. ß-elemene also increases the immunogenicity of cancer cells, makes tumor tissues sensitive to irradiation, reduces the proliferation of cancer cells, and induces the process of apoptosis in resistant tumors. 

*In vitro* and *in vivo* studies showed that ß-elemene makes cancer cells prone to radiation by inactivation of the ataxia telangiectasia mutated (ATM) signaling pathway which decreases the repair rate of damaged DNA. The formation of double strand break (DSB) activates ATM kinase following radiation. ß-elemene acts as an ATM inhibitor via the inhibition of phosphorylation of ATM after radiotherapy; so, it could cause increased the death rate by this way (13). Thus, ß-elemene causes radiosensitization via a reduction in the repair of double strand break (DSB) or an increase in radiation-induced DNA damage (17). Furthermore, recent studies have shown that radiation can increase the mRNA/protein expression of survivin in tumor cells and also increase HIF-1a activity. It hasbeen observed that tumors highly expressing survivinor HIF-1a are resistant to radiation. Previous studies have shown that ß-elemene enhances radiosensitivityof tumors by the inhibition of the survivin and HIF1a expression (18-21). It has been implicated that ßelemene induced radiosensitization is capable of the upregulation of and downregulation of Bcl-2 in cancer cells. It also activates caspase -7, caspase-9, and caspase -3, as well as inducing apoptosis in tumor cells and increasing the efficiency of radiotherapy (17). 

So, the study aimed to analyze the inhibitory effect ß elemene alone or in combination with radiotherapy on the human melanoma cell line (A375) using MTT test and flow cytometry. 

## Materials and Methods

The procedure of the study was approved by the Ethics Committee of the Iran University of Medical Science (No. IR. IUMS.REC1395.9311581001).

### Agents

ß-elemene was purchased from Abcam (Abcam, USA). Dulbecco’s modified Eagle’s medium (DMEM) and penicillin/streptomycin solution were procured from Atocel (Austria). Trypsin-ethylene diamine tetra-acetic acid (EDTA) and fetal bovine serum (FBS) inactivated with heat was purchased from Biowest company (France). 3-(4,5-dimethylthiazol-2-yl)-2,5diphenyltetrazolium bromide (MTT) and dimethyl sulfoxide (DMSO) were purchased from Merck (Germany) Annexin V/PI kit was purchased from Ebioscience company (CA).

### Cell culture conditions

A375 human melanoma cell line was purchased from the cellular bank of the Pasteur Institute of Iran (Iran) and then the cell culture was performed in standard conditions [37°C, 5% CO_2_, 1% antibiotic solution (pen-strep), high glucose DMEM containing 10% FBS]. 

### Cell proliferation assay

ß-elemene cytotoxicity and viability of incubated cells in different concentrations of the ß-elemene were evaluated using MTT assay. To perform this assay, cells were seeded at a density of 5000 cells/well (in 100 µl medium) into 96-well flat-bottomed microtiter plates at 24, 48, and 72 hours. During the incubation time, the medium was changed every other day. Then, the cells were incubated with different concentrations of ß-elemene (0-220 µg/ml) for 24 -72 hours with eight replicates for each treatment. Subsequently, cells were washed with phosphate buffer saline (PBS) after the treatment, and the medium was discarded. Afterwards, 10 µl of MTT dye (5 mg/ml in PBS) was added to each well; then, the plate was incubated for 3-4 hours at 37 C with 5% CO_2_. MTT-containing medium was removed, and formazan crystals dissolved by the addition of 100 µl DMSO to each well of the plate and kept in the dark place at 25°C for 15 minutes. Eventually, the absorbance of dissolved formazan was read at 570 nm using a microplate reader (DYNEX MRX, USA). The relative viability of A375 cells was described as the proportion of viable cells to untreated cells. The dose-response curves were plotted. The half maximal inhibitory concentration (IC_50_) value for ß-elemene was obtained from the dose-response curves by drawing log-linear regression and analyzed by the GraphPad Prism software version 6.01. 

### Irradiation

To perform radiotherapy, the A375 cell line was irradiated by using LINAC accelerator (Siemens, Germany), at the energy level of 6 MV at doses of 2 and 4 Gy. In order to reach the energy level of 6 MV, the distance between a radiation source and tissue surface should be 3cm. So, we placed 3 layers of Plexiglass (1 cm in diameter) under the plate and 5 layers (1 cm in diameter) above the plate. The irradiation process was applied in the front side at a distance of 100 cm from the bottom of the plate, and the radiation field size was 20×20 square centimeters. The monitor unit was calculated by the Core Plan Software in each irradiation process. 

### The combinatory effect of ß-elemene and radiotherapy

To examine the combinatory effects of ß-elemene and radiotherapy, A375 cells were cultivated in 96-well plates and incubated for 24 hours. After discarding the culture medium, ß-elemene were added at the concentrations of 40 and 80 µg/ml to each well. Next, the cells were incubated for 24 hours. The radiation was delivered at doses of 2 and 4 Gy X-ray at the energy of 6 MV. 

### Apoptosis analysis by Flow cytometry 

The rate of apoptosis was determined by Annexin V/ PI-Fluorescein isothiocyanate (FITC). The cells were treated with different ß-elemene concentrations (40, 80 µg/ml) for 24 hours and harvested by trypsinization after the treatment and centrifugation at 300 g for 5 minutes. After centrifuging, the cells were washed with 1X binding buffer and PBS. Then, the cells were suspended in 5 µl of Fluorochrome-conjugated and 1X binding buffer. Next, 100 µl of Annexin V was added into the cell suspension and incubated in a dark place for 20 minutes. The cells washed with 2 ml of binding buffer and resuspended in 200 µl of 1X binding buffer. Finally, 5 µl of propidium iodide (PI) staining solution was added to 200 µl cell suspension, and the samples were evaluated by flow cytometry (BD FACSCantoII, USA). 

### Statistical analysis

All the plotted data are shown as the mean ± SD, and the tests were at least repeated three times. Analysis of variance analysis (ANOVA) was conducted to analyze the data, and the comparison was made among different groups by the SPSS software version 16. The graphs and curves were assessed by the GraphPad Prism software (version 6.01). To indicate the significance of differences, the P<0.05 was statistically considered significant.

## Results

### Assessment of cell death and IC_50_ value of the drug following drug treatment using MTT assay

**Fig.1:**
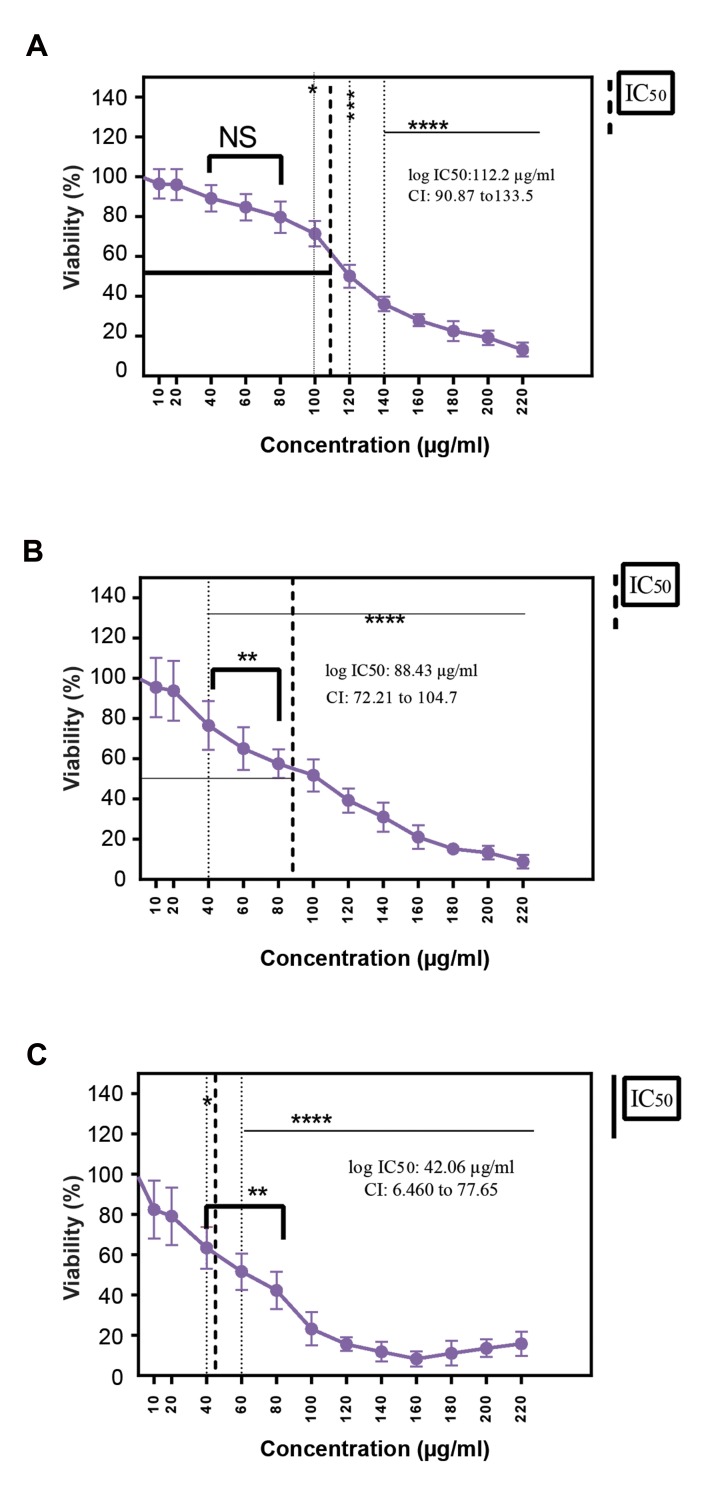
The growth rate of A375 cell line was inhibited by ß-elemene. A375 cells were cultivated in 96-well plates at a density of 5×103 cells/well and treated with different concentrations of ß-elemene in different time periods: **A.** 24, **B.** 48, and **C.** 72 hours. Cell proliferation was evaluated using the MTT assay. The IC_50_ value is a concentration of a drug that inhibits cell proliferation by 50% in comparison to the control. The data are shown as the means ± SD of three independent experiments. Asterisks indicate significant differences. ****; P<0.0001, ***; P<0.001, **; P<0.01, *; P<0.05, NS; Non significant, IC_50_: The half maximal inhibitory concentration, and CI; Confidence interval.

Cells were treated with various concentrations (0-220 µg/ml) of ß-elemene and incubated for 24, 48 and 72 hours. Figure 1A shows the percentage of cells viability after the 24-hour treatment process. Upon increasing the concentrations of ß-elemene from 10 to 80 µg/ml, the differences between cell viability do not significantly change. On the other hand, in a range of 100 to 220 µg/ ml ß-elemene a significant reduction in the viability of the cells was observed. After a 48-hour incubation period, as shown in Figure 1B, in cells treated with 10 µg/ml ß-elemene, a slight reduction in the viability of cells was observed (non-significant). At a concentration range of 20 to 220 µg/ml, the viability of cells was remarkably reduced. Figure 1C shows the viability of cells after a 72-hour incubation period. At a concentration range of 40 to 160 µg/ml ß-elemene, thecell viability was reduced significantly. In cells treated with 180, 200, and 220 µg/ ml ß-elemene, a slight increase in the viability of cells was observed. Also, no significant difference was observed at a concentration range of 10 and 20 µg/ml ß-elemene. All the treatment groups were compared with the control group (treatment-naive). Furthermore, the IC_50_ values for ß-elemene, at three different time points in human melanoma were calculated based on the results obtained from the MTT assay results. IC_50_ values for ß-elemene were 112.2 µg/ ml, confidence interval (CI): 90.87 133.5, 88.43 µg/ml, CI: 90.87 133.5 and (42.06 µg/ml, CI: 6.460 77.65) at 24, 48, and 72 hours, respectively. 

**Fig.2:**
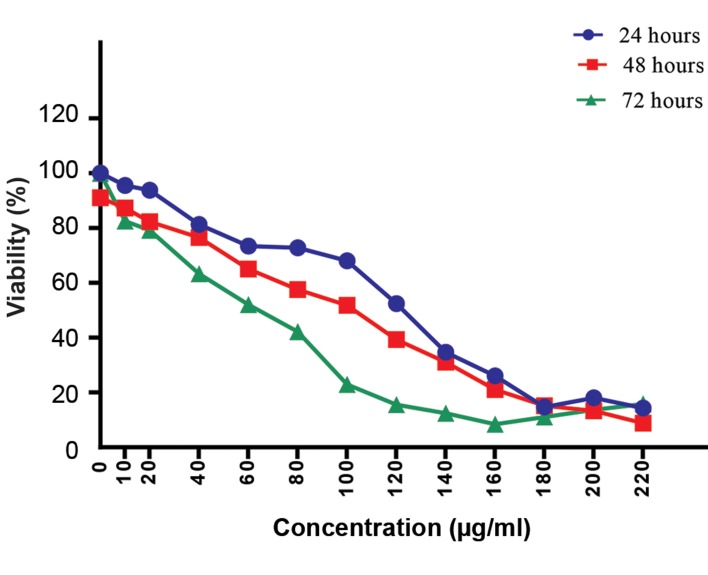
The capability of ß-elemene to inhibit cell proliferation was measured by the MTT assay. The viability of cells was approximately decreased in dose- and time-dependent manners. The difference in the IC_50_ value for ß-elemene was observed among the different incubation times. A significant reduction was detected in the viability of treated cells in a 72 hours incubation time compared with 24 and 48 hour periods. The data are presented as the means ± SD of three independent experiments. IC_50_; The half maximal inhibitory concentration.

Figure 2 shows the comparison of all the mentioned groups in three different times (24 hours, 48 hours, 72 hours). 

### Cell death evaluation in A-375 cell line, following the treatment with ß-elemene and radiation using the MTT assay

**Fig.3:**
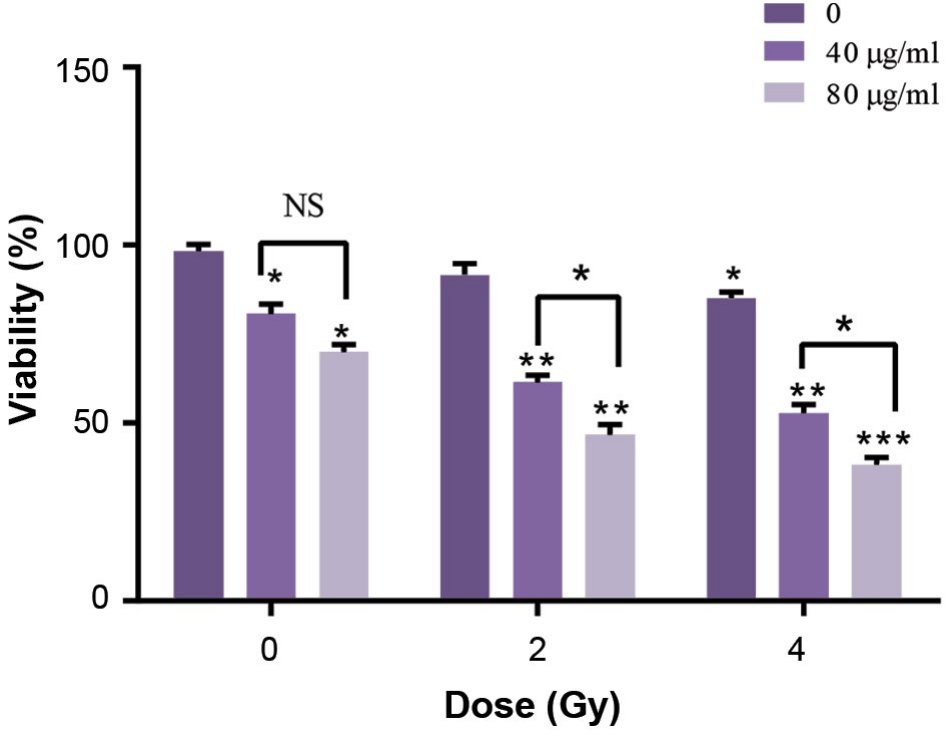
Cell proliferation was inhibited by ß-elemene. ß-elemene also increased the radiosensitivity of A375 cells. Comparison of the viability of A375 cells after the treatment with 40, 80 µg/ml of ß-elemene. After 24 hours of incubation time, cells were exposed to 2 and 4 (Gy) of 6 MV X-ray; then, the viability of cells was measured using the MTT assay. All treated groups were compared with the control group (treatment-naive). The data are presented as the means ± SD of three independent experiments. Asterisks indicate significant difference. *; P<0.05, **; P<0.01, ***; P<0.001, and NS; Non significant.

To study the effects of ß-elemene on radiotherapy, pretreating was performed on cells with two concentrations of ß-elemene, namely 40 and 80 µg/ml for 24 hours. Then, cells were exposed to radiation at doses of 2 and 4 Gy. Considering Figure 3, groups treated with a combination of ß-elemene and radiation, had a significant reduction in the viability compared with the groups treated with ß-elemene alone. Combination therapy with ß-elemene and radiotherapy significantly halted the proliferation of cancer cells compared with when each therapy was applied alone. 

**Fig.4:**
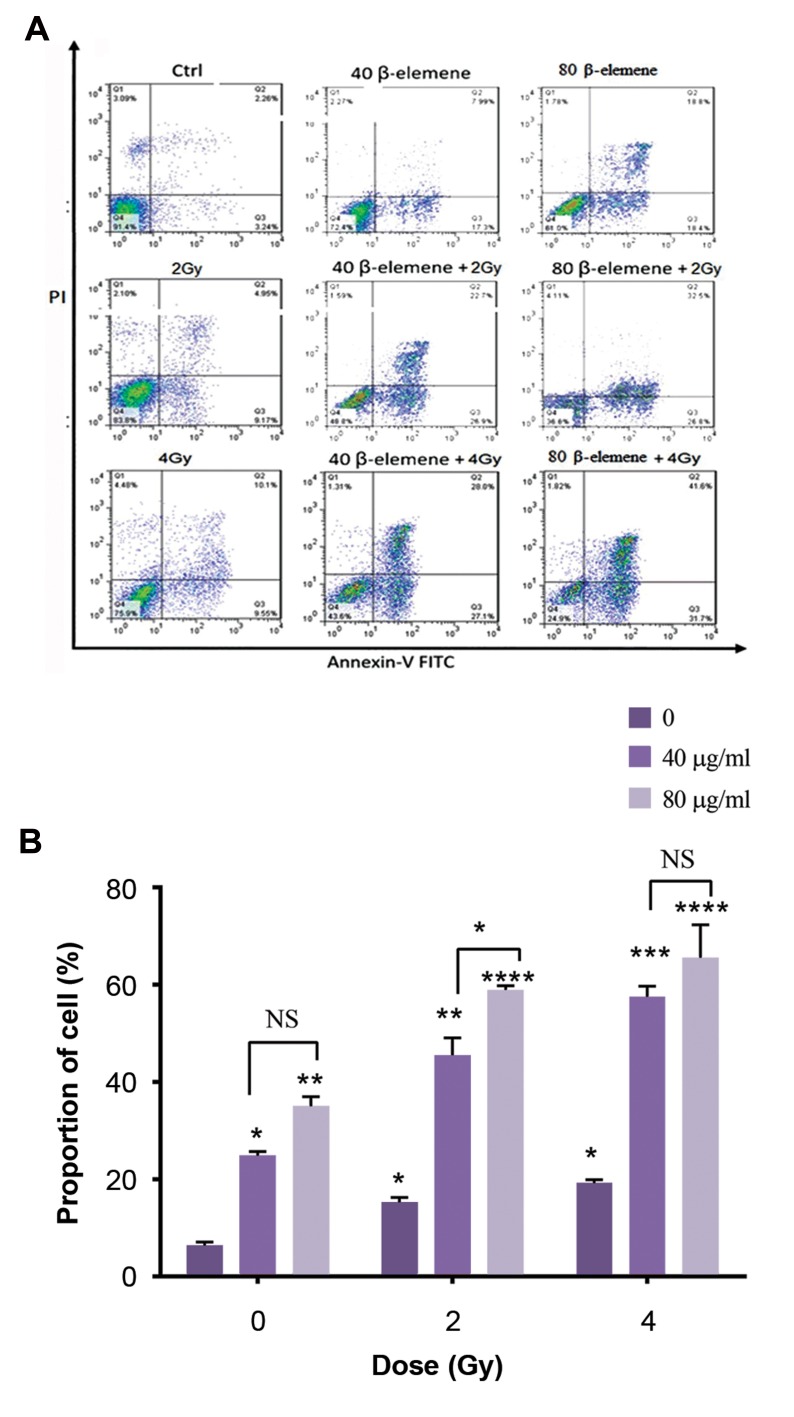
Annexin V-PI staining for the assessment of apoptosis following ß-elemene and radiation therapy in A375 human melanoma cell line. The pretreated process on cancer cells was performed at two concentrations of ß-elemene (40 and 80 µg/ml) for 24 hours. Then cells were exposed to 2 and 4 Gy irradiations in combination with ß-elemene for 24 hours. **A.** Early apoptosis was evaluated by Annexin V+/PI- staining, and Annexin V+/PI+ staining was applied as a marker for the detection of cells in the late apoptosis phase and **B.** PI and Annexin V double staining results indicated the induction of apoptosis by ß-elemene and enhanced radiation-induced apoptosis in human melanoma cancer cells. Cells were exposed to ß-elemene at concentrations of 40 and 80 µg/ml along with 2 and 4 Gy irradiations. All the treatment groups were compared with the control group (no drug). The data are presented as the means ± SD of three independent experiments. Asterisks indicate significant differences. ****; P<0.0001, ***; P<0.001, **; P<0.01, *; P<0.05, and NS; Non significant.

### The effect of ß-elemene on apoptosis of A375 cell line

According to the results, ß-elemene induces apoptosis and enhances the potency of the radiation driving A375 cancer cells to undergo apoptosis. Annexin V/PI staining was employed to detect the rate of apoptosis to show the effect of radiosensitization ability of ß-elemene on A-375 cell line. Following the treatment with ß-elemene, apparent morphological alterations were detected in cancer cells. Early apoptosis was examined via Annexin V+/PI- staining, while late apoptosis was monitored via Annexin V+/PI+ staining as depicted in Figure 4A. The quantification of different modes of cell death following ß-elemen and radiation exposure were shown in Figure 4B. The number of apoptotic cells in the groups treated with either ß-elemene or radiation were significantly higher than the control group (no therapy, no radiation). Furthermore, a significantly higher apoptotic rate was observed in the groups treated with radiation and ß-elemene at concentrations of 40 and 80 µg/ml (P<0.01, P<0.001, P<0.0001). The apoptotic rate was increased in parallel with an increment in the concentrations of ß- elemene. 

## Discussion

Melanoma is the most malignant and serious type of skin cancer (22). Patients suffering from melanoma can undergo various forms of therapy including surgery, chemotherapy, and radiotherapy, as well as receiving a combination of these treatment methods. Since melanoma tumor cells are among the most resistant cells to radiation (23); therefore, we need to novel treatments to conquer the resistance of this cancer to radiation. Recently, researchers attempt to find new anticancer drugs which among them radio sensitizers showed hold a great promise for the treatment of melanoma. ß-elemene, is a natural and traditional Chinese medicinal herb, indicating antitumor effects on many types of tumors with much fewer side effects (24). It has been demonstrated that ß-elemene could inhibit the growth and development of some chemotherapy-resistant tumors, including ovarian, prostate, and glioblastoma (14, 25). 

In this study, combination treatment with ß-elemene and radiation was examined to enhance radio sensitization with 6 MV X-ray in A375 cell line. The advantage of combination therapy is to increase the efficiency of the treatment when compared with standard treatment procedures. The MTT assay showed that ß-elemene could reduce the viability and inhibit the in-vitro growth of the human melanoma cell line in dose and time dependent manners. In the following step, after a 24hour incubation period, a significant reduction in the viability was observed when ß-elemene was applied at the concentrations range of 100 µg/ml to 220 µg/ml, however, after a 48-hour incubation period, treatment with ß-elemene at concentrations range of 20 µg/ml to 220 µg/ml reduced the viability of cells from 93 to 8%. The highest reduction rate of the cell viability was achieved at the concentrations range of 40 µg/ml to 160 µg/ml at a 72- hour incubation period. The trend of the reduction in the cell viability not only depends on the concentration of ß-elemene but also depends on the incubation time. The IC_50_ values obtained from the effect of ß-elemene on A375 cells were approximately 112.2, 88.43, 46.03 µg/ml at 24, 48, and 72 hours, respectively. These data indicate that ß-elemene vigorously decreases the viability of tumor cells. The statistical analysis of the data indicated a considerable reduction in the cells viability in the groups co-treated with ß-elemene and radiation compared with those treated with ß-elemene alone or the control group (no therapy). 

The cell viability of the group treated with 40 µg/ml of ß-elemene was 80%, while in combination treatment at a dose of 2 Gy with 6 MVX-ray reduced the viability to 61%. The results of the current study are consistent with previous studies. For example, Lu et al. (26) investigated the effect of ß-elemene on bladder tumor cells. They examined the cytotoxicity of ß-elemene using the MTT method and observed ß-elemene could inhibit the proliferation of T24 bladder carcinoma cells. Furthermore, Zhan et al. (27) evaluated the viability of human RCC 786-0 cell line after the treatment with different concentrations of ß-elemene for 24, 48 or 72 hours. The MTT assay indicated that ß-elemene inhibited the proliferation of 786-0 cells in dose and time depending manners. 

In this research, we analyzed the effect of ß-elemene on radiosensitivity of tumor cells to drive them to undergo apoptosis. The flow cytometry analysis indicates that ß-elemene is effective to induce apoptosis. ß-elemene induced apoptosis in A375 cell line was measured by Annexin V/PI staining. Treatment with either ß-elemene or radiation could somewhat increase the number of apoptotic cells in a dose-dependent way, confirming the results obtained from the MTT assay. Also, the flow cytometry analysis demonstrated that ß-elemene inhibits A375 cells proliferation and stimulates cell death by means of inducing apoptosis. The number of apoptotic cells by co-treatment with ß-elemene and radiation were significantly higher than those undergone cell death by the radiation or ß-elemene individually. For example, combination treatment with ß-elemene (40 µg/ml) and radiation at a dose of 4 (Gy) resulted in a decrease in the cell survival by 57.5% in comparison with the control. The percentages of apoptotic cells in response to the treatment of cells with 40 µg/ml ß-elemene or exposure to 4Gy of X-ray were 25/29% and 19/65%, respectively.

Liu et al. (28) investigated the effect of ß-elemene on stomach tumor cells using the flow cytometry method. They indicated a higher rate of apoptotic cells when incubated with ß-elemene in comparison with the control group. They found that ß-elemene interferes with the PI3K/Akt/mTOR/p70S6K1 pathways and causes apoptosis in tumor cells. Furthermore, the results of a study conducted by Dai et al. (29) showed that one of the important apoptotic pathways in tumors is the expression of Fas/FasL. ß-elemene is capable of inducing apoptosis in HepG2 cancer cells thereby the increase in the expression of Fas/FasL.

Li et al. (30) and Pugazhenthi et al. (31) have shown that ß-elemene could activate caspase-3 caspase-7, and caspase-9 and increase the ratio of Bax: Bcl-2, which is associated with the apoptosis of cancer cells. Also, Li et al. (32) observed that ß-elemene makes NSCLC cells sensitive to cisplatin triggering the intrinsic apoptosis pathway which involves Bcl-2 family proteins and inhibitor of apoptosis proteins (IAPs). So, our data showed that ß-elemene effectively enhanced radio sensitivity in A375 cell line. Similar results were obtained when the cells treated with 80 µg/ml of ß-elemene in combination with 2 and 4 Gy of X-ray. Liu et al. (33) investigated the effect of alone and also in combination with radiation on glioblastoma cells (U87-MG) using colony formation. In the colony formation assay, in cells treated with both ß-elemene and radiation, the colony formation ability was significantly reduced compared with the control group. As well, radiosensitivity was significantly enhanced following the treatment of the cells with ß-elemene. Li et al. (34) examined radiosensitization of ß-elemene in lung cancer cells (A549) by the comet assay and observed the same results. In general, ß-elemene increases tumor radio sensitivity through two mechanisms; i. The induction of cell cycle arrest at the G2/M phase and ii. The activation of ATM kinase by the DSB formation following radiation. So, the process of radio sensitization is related to the enhancement in radiation-induced DNA damage or a decrease in the repair of DSB (35). However, the precise mechanism of action of this herb is still unknown. 

## Conclusion

Radiation and ß-elemene are able to reduce the cell viability and increase apoptosis of melanoma cells. The cells viability was decreased by 23 and 30% for 2 and 4 Gy, respectively. Also, combination therapy with ß-elemene and radiation resulted in an increased rate of apoptosis. The percentages of apoptotic cells treated with 40 µg/ml ß-elemene and 4 Gy of X-ray alone were 25 and 19 %, respectively. The findings of this study indicated the efficiency of ß-elemene in treating melanoma cells and showed the necessity of more research in this field. 
